# CD10-positive mantle cell lymphoma: clinicopathologic and prognostic study of 30 cases

**DOI:** 10.18632/oncotarget.23571

**Published:** 2017-12-15

**Authors:** Jie Xu, L. Jeffrey Medeiros, Annapurna Saksena, Michael Wang, Jiehao Zhou, Jingyi Li, C. Cameron Yin, Guilin Tang, Lifu Wang, Pei Lin, Shaoying Li

**Affiliations:** ^1^ Department of Hematopathology, UT MD Anderson Cancer Center, Houston, TX, USA; ^2^ Department of Lymphoma and Myeloma, UT MD Anderson Cancer Center, Houston, TX, USA; ^3^ Department of Pathology, Indiana University, Indianapolis, IN, USA; ^4^ Department of Hematology, Tianjin First Center Hospital, China; ^5^ Department of Pathology, Henan Provincial People’s Hospital, Zhengzhou, Henan, China

**Keywords:** CD10, mantle cell lymphoma, pathology, prognosis

## Abstract

Mantle cell lymphoma is usually negative for CD10 which is useful in distinguishing MCL from other CD10 + B cell lymphomas. Here we assessed the clinicopathologic features of 30 cases of CD10+ MCL, the largest series to date in the English literature, and compared them with a group of 212 typical MCL cases (CD5+, CD10-negative, CD23-negative, cyclin D1+). The 30 patients with CD10+ MCL included 17 men and 13 women with a median age of 68 years. Compared with CD10-negative MCL, patients with CD10+ MCL showed a lower male predominance (*p* = 0.01), more often had a diffuse growth pattern (*p* = 0.04) and blastoid/pleomorphic morphology (*p* < 0.0001), and more often showed BCL6 expression (*p* = 0.009). In all MCL patients, CD10 expression was not associated with overall survival (OS) (*p* = 0.16). However, in more aggressive subsets of MCL patients including those with high Ki67 (> 60%), blastoid/pleomorphic morphology, or high MCL International Prognostic Index (MIPI), CD10 expression was associated with a worse OS (*p* = 0.003, 0.04, and 0.001, respectively). High Ki67 (> 60%), blastoid/pleomorphic morphology, and high MIPI were also been identified as poor prognostic factors patients with in CD10+ MCL (*p* = 0.001, 0.0003, and 0.01, respectively). In summary, CD10+ MCL more often has a diffuse growth pattern, blastoid/pleomorphic morphology, and BCL6 expression. In MCL patients with a high Ki-67 (> 60%), blastoid/pleomorphic morphology, or high MIPI, CD10 expression contributes to an even worse prognosis. MCL should be included in the differential diagnosis of CD10 + B cell lymphomas.

## INTRODUCTION

Mantle cell lymphoma (MCL) represents 3–10% of non-Hodgkin lymphomas and occurs predominantly in elderly men with a median age of 60 years. [1] Most patients with MCL present with stage III or IV disease and have an aggressive clinical course with a median survival of 3–5 years. [1] The genetic hallmark of MCL, t (11;14) (q13;q32) /*IGH-CCND1*, is present in > 95% cases and leads to overexpression of cyclin D1, a cell cycle regulator that facilitates dysregulation of the cell cycle at the G1-S phase transition. [1] Immunohistochemistry for cyclin D1 is an excellent surrogate for t(11;14) (q13;q32) /*IGH-CCND1* detected by conventional cytogenetic or FISH studies which is the current the gold standard for MCL diagnosis. However, ~5% MCL cases are cyclin D1-negative, a subset of which carries CCND2 translocations. [2] Recent studies have shown that SOX11 is highly expressed in most cases of MCL including cyclin D1-negative MCL, and is another useful diagnostic marker for MCL. [3]

Morphologically, classical MCL is characterized by a monomorphic proliferation of small to medium sized lymphocytes in a diffuse, nodular, or rarely mantle zone growth pattern. [1] However, a spectrum of morphologic variants is recognized, including blastoid, pleomorphic, small cell, and marginal-zone like variants. Most cases of MCL are believed to arise from a naïve pre-germinal center B cell and most MCL cases have a characteristic immunophenotype, positive for pan-B cell antigens, CD5, BCL2, and cyclin D1, and negative for CD23 and follicular center cell-associated antigens such as CD10 and BCL6. Therefore, CD10 is useful in distinguishing MCL from other CD10+ B cell lymphomas, mainly follicular lymphoma.

A few case reports and small case series of CD10+ MCL have been reported in the literature. [4–10] In this study, we describe the clinicopathologic features and outcome of 30 patients with CD10+ MCL and compare this cohort to a large group of MCL cases with a typical immunophenotype (CD5+, CD10-negative, CD23-negative, cyclin D1+).

## RESULTS

### Clinical findings

From a total of 794 patients with MCL accessioned in our files, 30 (3.8%) patients with CD10+ MCL were identified. The clinical and laboratory findings are summarized in Table 1. There were 17 men and 13 women with a median age of 68 years (range, 49–84 years) at the time of diagnosis. Twenty-one (70%) patients were 70 years of age or older. The most common physical finding was lymphadenopathy, identified in 19 (63%) patients. Bone marrow was involved in 16 of 25 (64%) patients and CNS was involved in 5 of 9 (56%) cases assessed. Five of 21 (24%) patients had elevated WBC count (all due to lymphocytosis): 4 of 4 (100%) further evaluated patients had peripheral blood involvement by MCL with 2 confirmed by flow cytometry and the other 2 confirmed by morphology. Nine of 20 (45%) patients showed an elevated serum LDH level. Most patients (19/20; 95%) who were fully staged at our hospital had stage III-IV disease. The Mantle Cell Lymphoma International Prognostic Index (MIPI) [11] was available in 16 cases: 7 patients had high, 8 patients had intermediate, and 1 had low MIPI scores.

**Table 1:** Clinical features of patients with typical CD10-negative and CD10+ MCL

	CD10-negative MCL (*n* = 212)	CD10+ MCL (*n* = 30)	*P* Value
Medium Age (yrs, range)	67 (29–95)	68 (49–84)	0.89
Age > 60 (yrs)^*^	77 (163/212)	70 (21/30)	0.49
Male:Female	5.1:1 (177/35)	1.3:1 (17/13)	**0.002**
Nodal Presentation^*^	50 (107/212)	63 (19/30)	0.33
BM Positive^*^	74 (145/196)	64 (16/25)	0.34
CNS Positive^*^	70 (7/10)	56 (5/9)	0.65
Elevated WBC^*^	17 (29/171)	24 (5/21)	0.54
Elevated Serum LDH^*^	26 (43/168)	45 (9/20)	0.11
Stage III or IV^*^	76 (144/190)	95 (19/20)	0.05
High MIPI^*^	23 (31/135)	44 (7/16)	0.12
Initial Chemotherapy^*^			0.47
Hyper-CVAD+/−R	55 (106/194)	54 (14/26)	
CHOP+/−R	20 (38/194)	27 (7/26)	
Other	15 (29/194)	19 (5/26)	
Initial CR^*^	83 (132/159)	69 (18/26)	0.11
With SCT^*^	16 (34/195)	15 (4/26)	1.0

^*^ = %(positive/evaluated); BM, bone marrow; CNS, central nervous system; WBC, white blood cells; LDH, lactate dehydrogenase; MIPI, Mantle cell lymphoma International Prognostic Index; Hyper-CVAD, hyperfractionated cyclophophsmide, vincristine, doxorubicin, dexamethasone, cytarabine and methotrexate; CHOP, cyclophosphamide, doxorubicin, vincristine, and prednisone; R, rituximab; CR, complete response; SCT, stem cell transplant.

In comparison to a control group of patients with CD10-negative MCL, the clinical features of the patients with CD10+ MCL were very similar; the only exception was that patients with CD10+ MCL showed a lower male predominance (male:female ratio of 1.3:1 vs 5.1:1 in typical CD10-negative MCL patients, *p* = 0.002).

### Pathologic findings

**Figure 1:**
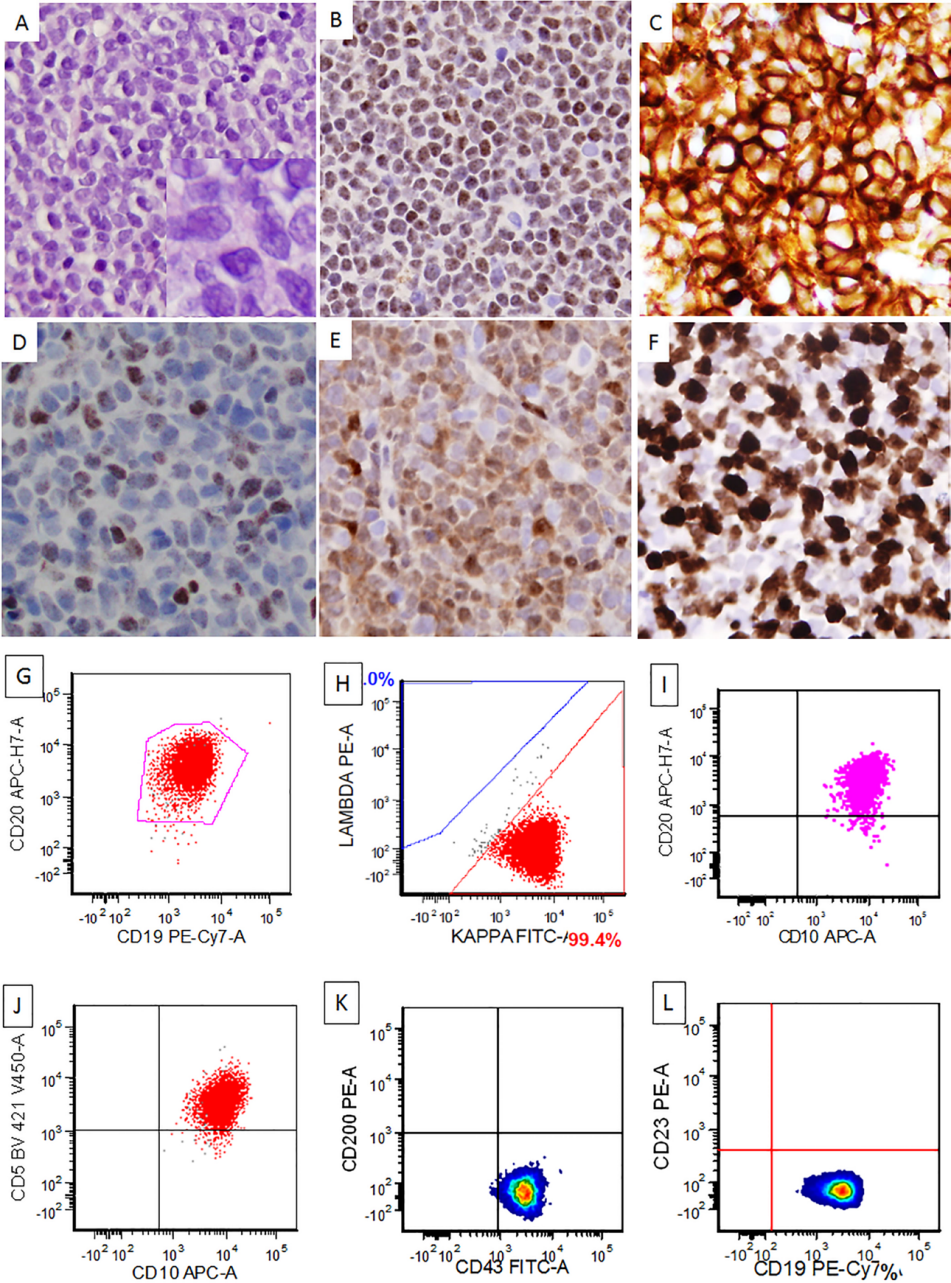
A representative case of CD10+ mantle cell lymphoma, blastoid variant The lymphoma cells grew in a diffuse pattern with blastoid morphology [(**A**) and insert, H&E, 400x and 1000x, respectively]. The lymphoma cells were PAX5+ (**B**), CD10+ (**C**), BCL6+ (subset, (**D**), Cyclin D1+ (**E**), and showed a high proliferation rate by Ki67 (**F**) (C–F), immunohistochemical stains, 400x). Flow cytometry study showed a kappa-restricted monoclonal B cell population that was positive for CD19, CD20, CD5, CD10, CD43, and negative for CD200 and CD23 (**G**–**L**).

In the 16 cases of CD10+ MCL with evaluable architecture, the lymphoma showed a nodular (*n* = 3; 19%), nodular and diffuse (*n* = 4; 25%), or diffuse (*n* = 9; 56%) pattern. The 30 cases of CD10+ MCL included 12 (40%) cases of classical MCL, 17 cases of blastoid MCL, and 1 case of pleomorphic MCL (Figure 1; Table 2). CD10 expression was evaluated by immunohistochemistry only in 10 (33%) cases, flow cytometry only in 10 cases, and by both immunohistochemistry and flow cytometry in 10 cases (Figure 1). CD10 detection by immunohistochemistry and flow cytometry was concordant in most of cases except 2: both being CD10+ by immunohistochemistry but negative by flow cytometry. Twenty-seven of 30 (90%) of cases were positive for CD5, and only 1 (4%) of 24 cases assessed was positive for CD23. Twenty-nine of 30 (97%) CD10+ MCL cases were positive for cyclin D1, and only 1 case was negative for cyclin D1 but this case showed t(11;14) by karyotype and FISH. SOX11 expression was detected in 12 of 19 (63%) CD10+ cases assessed. The proliferation index by Ki67 immunostain was assessed on 25 cases of CD10+ MCL with a medium Ki67 of 40%: 17 cases with Ki67 of > 30% and 8 cases with Ki67 of > 60%. BCL6 expression was detected in 6 of 19 (31%) CD10+ MCL and all 6 cases had Ki67 of < 60%. In the more aggressive CD10+ MCL (Ki67 > 60%, 8 cases), 3 cases were tested for BCL6 protein and all were negative. Conventional cytogenetic analysis was performed on 6 cases of CD10+ MCL and 4 (67%) of them showed complex karyotype but none had 8q24, 18q21, or 3q27 (*MYC, BCL2, or BCL6)* rearrangements. FISH for *MYC, BCL2*, and *BCL6* was available in 2, 2, and 1 case of CD10+ MCL, respectively, and all were negative for rearrangements. FISH for *CCND1* rearrangement was performed in 16 cases of CD10+ MCL and 88% (14/16) of them were positive. The 2 negative cases showed diffuse and strong cyclinD1 expression in one and SOX11 expression in the other.

**Table 2:** Pathological features of patients with typical CD10-negative and CD10+ MCL

	CD10-negative MCL (n = 212)	CD10+ MCL (n = 30)	P Value
Morphologic type^*^			
Classic	80 (169/212)	40 (12/30)	
Blastoid/Pleomorphic	20 (43/212)	60 (18/30)	**< 0.0001**
Immunohistochemistry			
Cyclin D1+^*^	100 (212/212)	97 (29/30)	0.12
SOX11+^*^	69 (42/61)	63 (12/19)	0.78
BCL6+^*^	7 (5/72)	31 (6/19)	**0.009**
Medium Ki67 (%)	25	40	
Ki67 > 30%^*^	49 (66/135)	68 (17/25)	0.08
Ki67 > 60%^*^	17 (23/135)	32 (8/25)	0.09

^*^ = %(positive/evaluated)

Compared with CD10-negative MCL, CD10+ MCL more often had a diffuse growth pattern [56% (9/16) vs 28% (35/124), *p* = 0.04], blastoid/pleomorphic morphology [60% (18/30) vs 20% (43/212), *p* < 0.0001], and showed BCL6 expression [31% (6/19) vs 7% (5/72), *p* = 0.009]. There were no other significant pathologic or immunophenotypic differences beween the two groups (Table 2).

### Treatment and response

Twenty-six patients with CD10+ MCL had available information about treatment and clinical follow up. Patients with CD10+ MCL were treated with different chemotherapy regimens over the time interval of this study, with or without stem cell transplant (SCT). Fourteen (54%) patients were treated with hyperfractionated cyclophosphamide, vincristine, doxorubicin, dexamethasone, cytarabine and methotrexate (Hyper-CVAD), and 7 (27%) patients were treated with cyclophosphamide, doxorubicin, vincristine, and prednisone (CHOP), with or without rituximab (in earlier years) (Table 1). After initial induction chemotherapy, 18 (69%) patients achieved complete remission. Four of 26 (15%) patients received SCT: 3 autologous, and 1 patient received autologous followed by allogeneic. There was no significant difference in the treatment and initial complete remission rate between patients with CD10+ MCL versus patients with CD10-negative MCL.

### Prognosis

**Figure 2:**
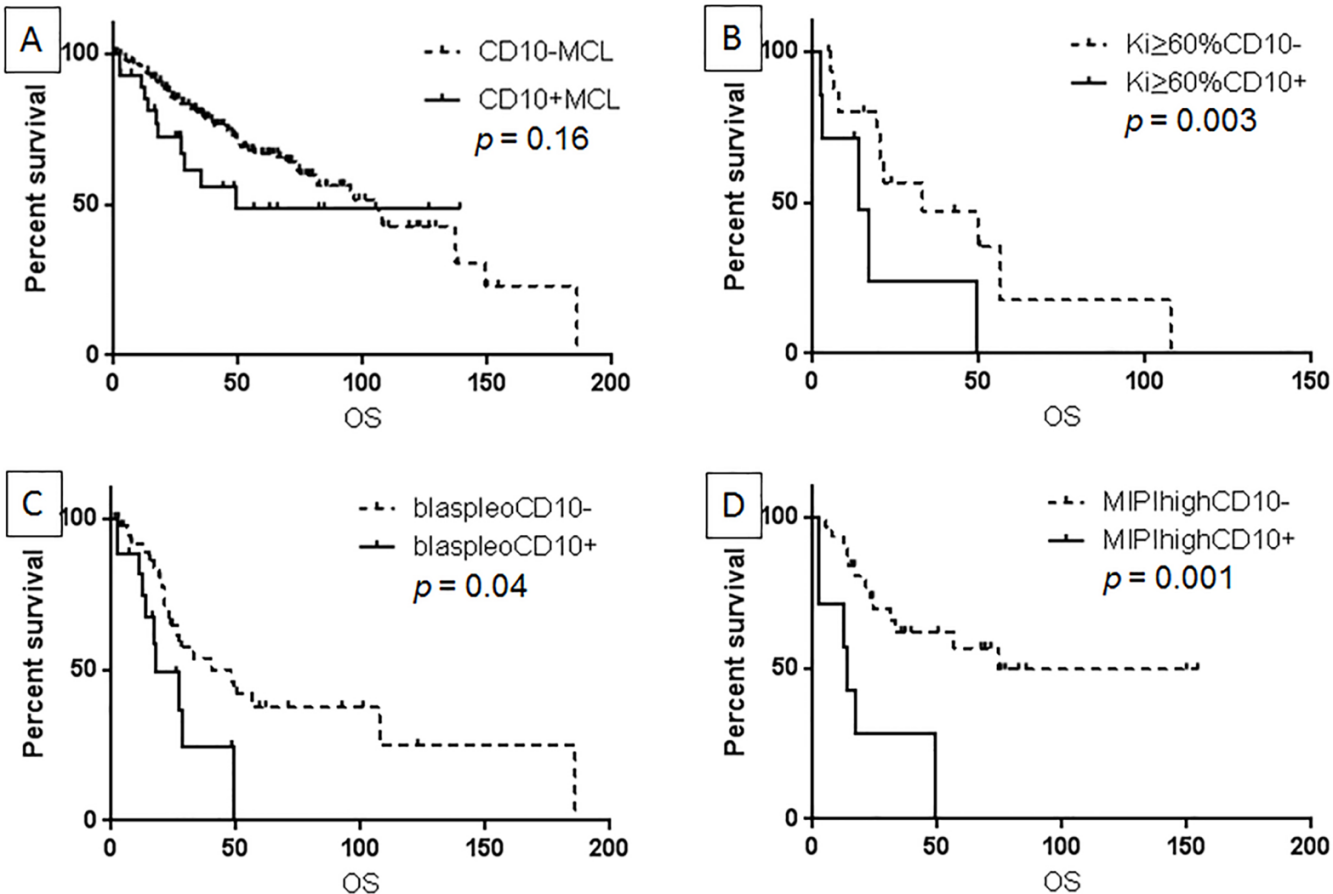
CD10 expression was not associated with overall survival (OS) in all MCL patients (**A**), but predicted a worse OS in patients with Ki-67> 60% (**B**), patients with blastoid/pleomorphic morphology (**C**), and in patients with high MIPI (**D**).

After a median follow up of 25.4 months, 11 (36.7%) patients died. The median overall survival (OS) for patients with CD10+ MCL was 49.4 months, not significantly different from patients with CD10-negative MCL (Figure 2A, *p* = 0.16). The comparison was further studied in more aggressive subsets of MCL. In MCL patients with Ki67 (≥ 60%), blastoid/pleomorphic morphology, or high MIPI, CD10 expression was associated with a worse OS (*p* = 0.003, 0.04, 0.001, respectively) (Figure 2B–2D). In contrast, in MCL patients with lower Ki-67 (< 60%), classic morphology, or low-intermediate MIPI, no significant difference in OS was observed between patients with CD10+ versus CD10-negative MCL (*p* > 0.05 for all).

**Figure 3:**
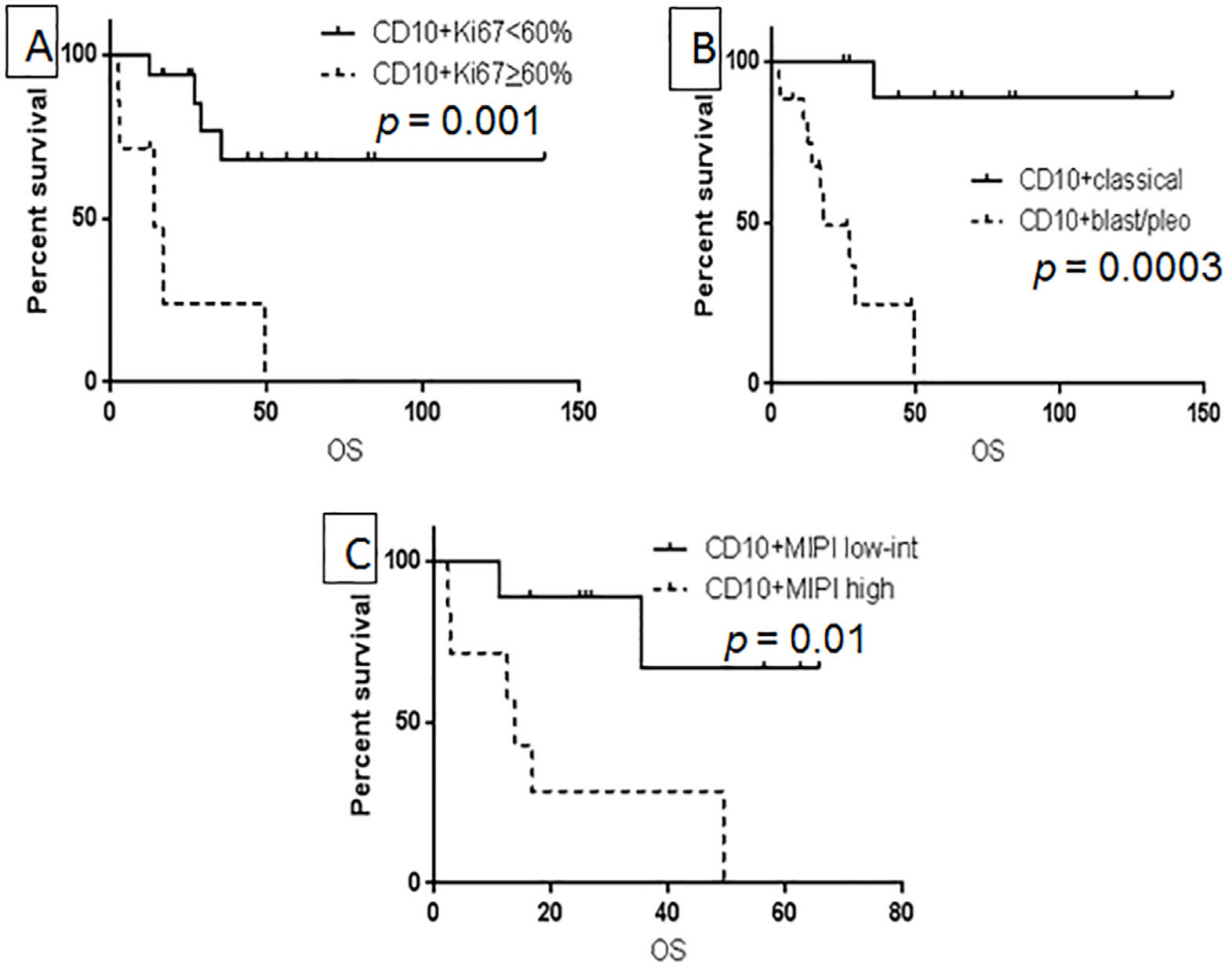
Prognostic factors associated with OS in CD10+ MCL: Ki-67 using 60% as cutoff value (**A**), blastoid/pleomorphic type (**B**), and high MIPI (**C**).

The prognostic significance of Ki67, blastoid/pleomorphic morphology, and MIPI score were also examined in CD10+ MCL patients. Ki67 predicted OS only when the cutoff of > 60% was used (Figure 3A) and the generally used 30% cutoff was not associated with OS in CD10+ MCL patients (*p* = 0.15). Both blastoid/pleomorphic morphology and high MIPI were associated with a poor prognosis in patients with CD10+ MCL (*p* = 0.0003 and 0.01, respectively) (Figure 3B–3C). Overall survival was further compared between patients with SOX11+ versus SOX11-negative CD10+ MCL and there was no significant difference in OS between the two groups (*p* = 0.8).

## DISCUSSION

The CD10 antigen is a 110-kd transmembrane glycoprotein that is normally expressed in early lymphoid progenitors and normal germinal center B cells. Assessment of CD10 expression by flow cytometry or immunohistochemistry has been widely used in the diagnostic work-up of B cell lymphomas. CD10 is usually expressed in B cell lymphomas of follicular center cell origin, such as follicular lymphoma, Burkitt lymphoma, a subset of diffuse large B cell lymphoma, and most cases of lymphoblastic lymphoma/leukemia. However, expression of CD10 has been detected occasionally in other B cell lymphomas that usually do not derive from follicular center B cells, such as MCL, extranodal marginal zone lymphoma, and chronic lymphocytic leukemia/small lymphocytic lymphoma. [6, 7]

Information regarding the frequency and clinical importance of CD10 expression in MCL is very limited in the literature. Most studies of CD10+ MCL were case reports and rarely small case series. [4–9] In a study focused on CD5+CD10+ B cell lymphomas, Dong et al reported 9 cases of CD10+ MCLs. [6] Gao et al showed that 4 of 50 (8%) MCL cases expressed CD10 and there was no significant difference in Ki67 rate between MCL with a variant immunophenotype and MCL with a typical immunophenotype. [9] Gualco et al reported that 3 of 127 (2.4%) cases of MCL had CD10+ tumor cells. [12] Pizzi and colleagues recently reported CD10 expression in 11 of 165 (6.7%) cases of MCL and showed a correlation with female gender, MUM1/IRF4 expression and higher Ki67 index. [12a] In this study, about 4% of MCLs were positive for CD10 expression.

So far, in the literature, only one study compared CD10+ MCL with CD10-negative MCL and there were no significant differences in clinicopathological features or outcome between them. [10] However, this study was a small case series consisting of only 9 cases of CD10+ MCL. Therefore, it is still unclear whether CD10+ MCL has distinctive clinicopathologic features or prognostic significance. In this study, we described the clinicopathologic features and prognosis of 30 cases of CD10+ MCL, the largest series to date in the English literature. We found that the clinical features of patients with CD10+ MCL were similar to the patients with CD10-negative MCL, with the exception that the male-to-female ratio was much lower in CD10+ MCL patients.

In this study, CD10+ MCL more frequently had a diffuse growth pattern and more often exhibited blastoid/pleomorphic morphology than CD10-negative MCL. Limited studies have suggested that CD10 expression in MCL is associated with blastoid morphology. [5, 6, 8] A leukemic MCL case was reported composed of both classical and blastoid components: the classical component had the typical immunophenotype of MCL (CD10-negative), whereas the blastoid component was CD10+. [5] Zanetto et al reported 4 blastoid MCLs that transformed from classical MCL and 2 of these cases acquired CD10 expression at time of transformation, [8] and a similar case was observed by Yin and colleagues [13]. In this cohort, all 30 patients with CD10+ MCL had CD10 expression at time of initial diagnosis. The mechanisms underlying the association between CD10 and blastoid/pleomorphic MCL is unknown and needs to be further investigated.

The immunophenotype of CD10+ MCL cases is similar to that of typical CD10-negative MCL with the exception of BCL6 expression. BCL6, a germinal center-associated antigen, is a transcription factor and master regulator of B cell differentiation in germinal center B cells and thus is characteristically found in B cell lymphomas of germinal center origin. BCL6 expression has been reported in CD10+ MCL in the literature with a positive rate ranging from 11% to 75%, though most studies consisted of a small number of cases. [4, 8–10] In one relatively large series of MCL study, BCL6 expression was observed in about 12% (15 of 127 cases) of MCL. [12] In the current study, BCL6 was positive in about 31% (6/19) of CD10+ MCL, substantially higher than in CD10-negative MCL (5/72, 7%; *p* = 0.009). CD10 expression in MCL, together with BCL6 in a subset of cases, raised the possibility that this small subset of MCL might be derived from germinal center B cells. Analysis of somatic mutation of immunoglobulin heavy chain variable region genes (*IGHV*) may help to address the issue. Historically, naive pre-germinal center mantle zone B cells have been considered as the normal counterpart to MCL cells based on expression of CD5 and IgM/IgD, and early descriptions of most MCL with unmutated *IGHV*. However, recent studies have shown about 20-30% of MCL cases carry somatic mutations of *IGHV* gene, implying that the neoplastic cells in these cases either have been exposed to the germinal center environment or, alternatively, that somatic hypermutations have been acquired in a non-germinal center context. [14–17] It is reasonable to hypothesize, as has been done by others, that: (1) in the absence of *IGHV* mutations, MCL may be derived from naïve B cells; (2) MCL carrying a high mutational load may originate from cells strongly influenced by the germinal center microenvironment; (3) the progenitor cells of cases with a low number of somatic mutations may derive from cells of the marginal zone, intermediate cells between naïve and germinal center cells. [18] Whether CD10+ MCL arises from germinal center B cells is controversial. A study by Zanetto et al studied 5 cases of CD10+ MCL expressing BCL6 protein and found that 1 case carried a *BCL6* translocation and 3 others had extra copies of the *BCL6* gene, suggesting the BCL6 protein expression found in these cases could be the result of chromosomal alterations involving *BCL6*, rather than resulting from a germinal center origin of the lymphoma. Only 1 of these 5 cases of CD10+ MCL showed somatic mutations, at a lower level than typically seen in germinal center B-cell lymphomas, indicating that they did not arise from germinal center B cells. [8] In contrast, a recent study compared the gene expression profile of CD10+ MCL with CD10-negative MCL; the former showed a distinctive germinal center B-cell signature in CD10+ MCL, supporting a germinal center origin. [10]

The most consistently reported adverse prognostic factors in MCL patients are high MIPI score, high Ki67 index, and, in some studies, blastoid/pleomorphic morphology. Whether these factors also predict survival in CD10+ MCL patients has not been reported in the literature. In this CD10+ MCL cohort, patients with a high MIPI score had worse overall survival than patients with a low/intermediate MIPI score. The Ki67 index is another independent prognostic factor for MCL. In our study, the medium Ki67 was 25% in CD10-negative MCL and 40% in CD10+ MCL, respectively. The medium Ki67 of 25% in our CD10-negative MCL is slightly higher than the 20% reported in a previous study. [19] However, 80% of our cases had classic morphology and 20% had blastoid/pleomorphic morphology. The percentage of blastoid/pleomorphic MCL in our study is much higher than the above literature (20% vs 10%) which may explain the slightly higher medium Ki67 value in our study. The cutoff value for Ki-67 has varied between studies: 30%, 40%, 60%, etc. [19–21] Others have divided MCL patients into 4 proliferation groups, with Ki67 < 20%, 21–40%, 41–60%, and > 60%, and their median survival times were 53 months, 33 months, 19 months, and 13 months, respectively (*p* < 0.001). [21] Recent results from Randomized Trials of the European MCL Network showed that Ki67 index was superior to cytology (classical vs. blastoid) and growth pattern as a prognostic factor in MCL; and patients with Ki67 > 30% had inferior outcome compared with patients with Ki-67 < 30% in both blastoid and classical MCL. [19] In our CD10-negative MCL cohort, the survival difference was observed when using both 30% and 60% as cut off (*p* < 0.0001 and *p* = 0.0002, respectively; data not shown), consistent with those reported in the literature. In contrast, in CD10+ MCL patients, a survival difference was only significant when using a 60% cutoff, suggesting 60% was the best cutoff value for Ki67 in predicting prognosis among patients with CD10+ MCL. Blastoid/pleomorphic morphology has been reported to be associated with a more aggressive clinical course and shorter median survival. [22, 23] Recent results from Randomized Trials of the European MCL Network showed that patients with blastoid MCL had a shorter OS, independently of MIPI score, compared with patients with non-blastoid MCL. However, multivariable Cox regression showed that the prognostic effect of blastoid cytology was largely explained by the Ki67 index, which was generally higher in blastoid MCL. [19] In this cohort of CD10+ MCL cases, blastoid/pleomorphic morphology was associated with an inferior outcome.

Another potential prognostic marker for MCL is SOX11 expression. SOX11, a neuronal transcription factor, was identified as a very specific marker of MCL. [24] The positive rate of SOX11 in MCL varies in the literature, ranging from 69% to 98%, regardless of cyclin D1 status. [3, 25–28] Although some recent studies suggest that SOX11 expression may be a predictor of poor outcome in MCL, its prognostic role is still controversial. [3, 28–30] SOX11 expression has not been previously studied in CD10+ MCL. In this study, SOX11 was detected in 63% of CD10+ MCL, similar to the CD10-negative MCL group (69%, *p* = 0.78). We also found no association between SOX11 expression and OS in the CD10+ MCL cohort.

Our study showed no significant difference in OS between patients with CD10+ versus CD10-negative MCL, similar to a previous study. [10] We found, however, that CD10 expression predicted a worse outcome in more aggressive subsets of MCL patients, such as those with Ki67 > 60%, blastoid/pleomorphic morphology, or a high MIPI score.

Although not uncommon, CD10+ MCL needs to be considered in the differential diagnosis of CD10+ B cell lymphomas. Recognizing CD10+ MCL is particularly important given the fact that MCL is much more aggressive than all other small B cell lymphomas and usually need more intensive treatment. CD10 expression in MCL can lead to considerable diagnostic difficulty, especially when CD5 is negative as was observed in 10% of CD10+ MCL cases in this study. CD10 expression by MCL may lead to misdiagnosis as follicular lymphoma in the case of small cell or classical morphology, germinal center B-cell-like diffuse large B cell lymphoma in the case of pleomorphic morphology, or B lymphoblastic lymphoma/leukemia in the case of blastoid morphology. Assessing cyclin D1 and/or SOX11 expression by immunohistochemistry, or *CCND1* rearrangement by conventional cytogenetic or FISH studies will help to rule out CD10+ MCL and avoid misdiagnosis.

In conclusion, we studied a large cohort of CD10+ MCL and our data suggested that CD10+ MCL had some distinctive features compared with CD10-negative MCL. Patients with CD10+ MCL were more likely to be women, more often had a diffuse growth pattern and blastoid/pleomorphic morphology, and more often expressed BCL6. CD10 expression was associated with a worse overall survival in more aggressive MCL, including patients with a high MIPI score, high Ki-67 (> 60%), or blastoid/pleomorphic morphology.

## MATERIALS AND METHODS

### Case selection

We searched the database of the Department of Hematopathology at The University of Texas MD Anderson Cancer Center from January 1, 2004 to December 31, 2016 for cases of MCL that were positive for CD10 as shown by flow cytometry analysis or immunohistochemistry. A large group of MCL cases with a typical MCL immunophenotype (CD5+, CD10-negative, CD23-negative, cyclinD1+) was selected as a comparison group. The diagnosis and subclassification were based on the criteria as specified in the World Health Organization classification. [1] The diagnosis of MCL has been confirmed by immunohistochemical stain of cyclin D1 and/or SOX11, and/or t(11;14) (q13;q32) by conventional cytogenetic or *CCND1* rearrangement by FISH studies. Clinical information was obtained by review of medical records. This study was approved by the institutional review board.

### Immunophenotypic analysis

Immunohistochemical studies were performed using formalin-fixed, paraffin-embedded (FFPE) tissue sections either at the time of diagnosis or retrospectively for this study. Immunohistochemical analysis was performed on an automated immunostainer (Leica Bond-Max IHC Stainer, San Diego, CA). The 4-μm-thick FFPE tissue sections were deparaffinized and underwent heat-induced antigen retrieval using the Bond Max Epitope Retrieval 1 solution for 15 minutes. The sections were incubated with an antibody against a specific antigen. The Bond Refine Polymer detection system was used for visualization. The antibodies used were specific for CD3, CD20, BCL-6, and Ki67 (Dako, Carpinteria, CA, USA); CD5 and cyclin D1 (SP4; Labvision/Neomarkers, Fremont, CA, USA); CD10, CD23, and BCL2 (Novocastra/Vision Biosystem, Benton Lane, Newcastle-upon-Tyne, UK); PAX5 (Transduction Labs, San Diego, CA, USA), and SOX11 (Cell Marque, Rocklin, California, USA). The positive cutoff was ≥ 30% for CD10 and BCL6 and > 10% for SOX11. [27, 31] The Ki67 index was calculated as the percentage of positive cell nuclei of total, recorded in 5% increments. Residual reactive germinal centers and areas of dense T cells were excluded.

Flow cytometry immunophenotypic analysis was performed on cell suspensions of tissue biopsy specimens or bone marrow aspirates using either a FACScanto II or FACSCalibur cytometer (Becton-Dickinson Biosciences, San Jose, CA, USA). Lymphocytes were gated for analysis using side scatter versus forward scatter and CD45 expression versus side scatter. The panel of monoclonal antibodies included reagents specific for CD3, CD5, CD10, CD11c, CD19, CD20, CD22, CD23, CD30, CD38, CD43, CD45, CD79b, CD200, FMC-7, and surface immunoglobulin kappa and lambda light chains (Becton-Dickinson Biosciences, San Jose, CA, USA).

### Conventional cytogenetic analysis and fluorescence *in situ* hybridization

Conventional cytogenetic analysis was performed on metaphase cells prepared form bone marrow aspirates or cell suspensions from tissue biopsy specimens as previously described. [32] Twenty Giemsa-banded metaphases were analyzed, and the results were reported using the International System for Human Cytogenetic Nomenclature (2016). Fluorescence *in situ* hybridization (FISH) analysis for detection of *CCND1* rearrangement was performed using a Vysis LSI *IGH/CCND1* dual color, dual fusion translocation probe on interphase nuclei obtained from bone marrow cells or tissue sections, according to the manufacturer’s instructions (Vysis/Abbott Laboratories, Des Plaines, IL, USA).

### Statistical analysis

Statistical analyses were performed using the Graph-Pad Prism 6. Fisher’s exact test was utilized to compare the clinicopathologic features between patients with CD10+ MCL and patients with typical MCL. Overall survival (OS) was calculated from the date of initial diagnosis to the date of death or last follow-up. Survival was analyzed using the Kaplan–Meier method and was compared using the log rank test. A *p* value of less than 0.05 was considered statistically significant.
